# Trace Elements Speciation of Submicron Particulate Matter (PM1) Collected in the Surroundings of Power Plants

**DOI:** 10.3390/ijerph121013085

**Published:** 2015-10-16

**Authors:** Elwira Zajusz-Zubek, Konrad Kaczmarek, Anna Mainka

**Affiliations:** 1 Department of Air Protection, Silesian University of Technology, 22B Konarskiego St., Gliwice 44-100, Poland; E-Mail: Anna.Mainka@polsl.pl; 2 Institute of Mathematics, Silesian University of Technology, 23 Kaszubska St., Gliwice 44-100, Poland; E-Mail: Konrad.Kaczmarek@polsl.pl

**Keywords:** PM1, trace elements, power plants, chemical fractionation, enrichment factor (EF), principal component analysis (PCA)

## Abstract

This study reports the concentrations of PM1 trace elements (As, Cd, Co, Cr, Hg, Mn, Ni, Pb, Sb and Se) content in highly mobile (F1), mobile (F2), less mobile (F3) and not mobile (F4) fractions in samples that were collected in the surroundings of power plants in southern Poland. It also reports source identification by enrichment factors (EF) and a principal component analysis (PCA). There is limited availability of scientific data concerning the chemical composition of dust, including fractionation analyses of trace elements, in the surroundings of power plants. The present study offers important results in order to fill this data gap. The data collected in this study can be utilized to validate air quality models in this rapidly developing area. They are also crucial for comparisons with datasets from similar areas all over the world. Moreover, the identification of the bioavailability of selected carcinogenic and toxic elements in the future might be used as output data for potential biological and population research on risk assessment. This is important in the context of air pollution being hazardous to human health.

## 1. Introduction

One of the most dangerous pollutants in atmospheric air is the particulate matter (PM). Ambient particles significantly influence air quality due to their impact on visibility and climate change [1,2]. The major anthropogenic sources of fine particles are the combustion processes of fossil fuels [3].

Scientists have become increasingly interested in the fate of potentially toxic trace elements in coal-fired power plants [4,5,6]. These elements, namely As, Be, Cl, Cr, Cd, Co, Hg, Mn, Ni, Pb, Sb and Se have been defined as being hazardous to humans (Hazardous Air Pollutants (HAPs) [7]. The International Agency for Research on Cancer (IARC) classified these elements to one of the following categories. Group 1: agents carcinogenic to humans—As, Be, Cr, Cd and Ni; group 2: agents probably carcinogenic to humans—Cl, Co and Pb; and group 3: Not classifiable as to its carcinogenicity to humans—Se [8]. Hg, Mn [9] and Sb are toxic elements. Sb is also suspected to be carcinogenic [10].

All of the power plants in Poland are currently equipped with electrostatic precipitators with high retention efficiency (>99.9%). Fly ash escaping the electrostatic precipitators of the power plants is now a minor contributor to the ambient total suspended particulates (TSP) levels. The penetration of fine particles may still be as high as 15%, due to their low charging efficiency [11,12]. According to the high rate of coal, consumption problems that are connected with air quality in the vicinity of coal combustion sources will intensify [13,14].

Worldwide industry and energy production that are based on coal are substantial sources of air pollution. Other sources with significant contributions were found, namely municipal and traffic emissions, domestic coal burning, vegetative burning (wood combustion and agricultural burns), open-air burning of refuse and re-suspension of dust.

In the coal combustion process, there are three streams of general tendencies in the distribution of trace elements: Solid waste, fly ash and waste gas. The streams are influenced by the properties of trace compounds and the efficiency of dust control systems [15]. Other studies [15,16,17] have revealed that, among the trace elements that are emitted in power plants, the fly ash that is formed during coal combustion processes mainly contains Cr, Cu, Mn, Ni, Pb and Zn.

PM1 is deposited in the alveolar regions of the lungs. The efficiency of their adsorption is 60%–80% [18]. Many epidemiological and toxicological studies have reported a link between PM exposure and decreased lung function, aggravation of respiratory diseases and increased hospitalization admissions for the elderly [5,19,20]. A chemical analysis of PM as the indicator of pollution sources may provide new insights into the underlying relationship between PM air pollution and health. Toxicological studies have suggested that the determination of the total trace element content of airborne particulate matter is a poor indicator of their bioavailability, mobility and toxicity [21]. Bioavailability is the prime consideration in environmental risk assessment of toxicity. An evaluation of the potential toxicities of trace elements is based on the distributions of their chemical forms. The bioavailability of elements depends on the characteristics of their surfaces, strength of their bonds and properties of solutions in contact with particulate matter [18]. The amount of potentially bioavailable trace elements in PM can be estimated from the water-soluble fraction and the fraction extracted by a dilute salt by extracting. Thus, investigations of the soluble or extractable elements in PM have attracted much more attention in this field of study. The chemical mobility of elements determined by leaching procedures is a good indicator for their bioavailability, namely the degree and rate that a substance is absorbed into a living system or is made available at the site of physiological activity.

Partial and sequential extractions are commonly used to determine elemental associations with various fractions. Some fractionation schemes for airborne particulate matter (APM) were summarized in a review of Smichowski *et al.* [18]. For our work, we derived a modified Tessier’s scheme of chemical extraction optimized by Fernandez-Espinosa *et al.* [22,23]. This extraction scheme was selected because it has been optimized for filter-collected fine urban particles and provides conditions that are more similar to the deposition and solubilization in the human lung. The scheme (Table 1) distinguishes four fractions depending on the element’s mobility. The first fraction (F1) is water-soluble—highly mobile; the second (F2) is bound to carbonates and oxides and reducible metals—mobile; the third (F3) is bound to organic matter, oxidizable metals and sulphides—less mobile; and the fourth (F4) is residual, permanently connected with minerals—not mobile. A quantitative evaluation of these forms—a fractionation analysis—is currently one of the most efficient methods to enable the prediction of the conditions in which ecosystem contamination can occur.

The availability of scientific data concerning chemical composition of PM1, including fractionation analyses of trace elements, remains very limited. This is a result of the technical difficulties of an analysis, sample collection and also, more recently, a lack of detailed definition of the scientific aims of fractionation research.

Specifically, this paper includes the following aspects: (1) a comparative analysis of the PM1 levels in the surroundings of four individual working power plants; (2) a chemical fractionation of the trace elements (As, Cd, Co, Cr, Hg, Mn, Ni, Pb, Sb and Se) in PM1, collected from areas near the selected power plants; (3) a statistical analysis of the correlation coefficients to illustrate the relationship among the trace elements, as well as the enrichment factors (EF); and (4) a principal component analysis (PCA) to identify the possible sources of these elements in this area.

## 2. Material and Methods

### 2.1. Sampling Sites

In order to eliminate the influence of the heating season, especially domestic and municipal emissions, the sampling campaigns were only carried out during the summer season. Samples were collected from 28 May to 23 September 2014 (16 weeks) in the surroundings of four working power plants fired with hard coal. The sampling campaign was divided into four sessions, separately for every point. In order to collect sufficient amount of PM1, each session was performed continuously for seven days. Four sessions were conducted at each point. Totally there were 16 samples and 16 blank filters (stored in sampling area but not exposed). All of the sampling points were located in the north-east of the selected power plants, according to the dominant wind direction (207°) in the region. The distances of the points from the corresponding power plants were approx. 2 km. The choice of a certain location was dependent on the possibility of locating measuring apparatus at a particular estate with permission from the owners.

**Figure 1:**
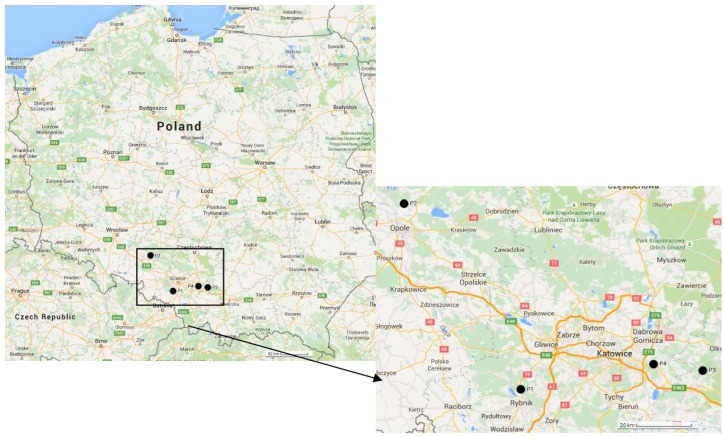
Localization of sampling sites in Southern Poland (Map data: ^©^2015 Google, ORION-ME).

The sampling points were located in southern Poland. Three of them (P1, P3, P4) are situated in the industrial macro-region of Upper Silesia, while P2 is situated approx. 70 km to the west from the macro-region. Figure 1 shows the study area and the sampling locations in the surroundings of the selected four power plants. The first point, labelled P1 in Golejów (50°08ʹ37.87ʹʹ N; 18°32ʹ15.76ʹʹ E), is situated in a peri-urban area of Rybnik in the surroundings of a power plant with an installed capacity of 1775 MW. Location P1 is inhabited by 2300 residents. The second point, labelled P2 (50°45ʹ35.41ʹʹ N; 17°56ʹ20.43ʹʹ E), is located at the rural site of Świerkle village in the surroundings of a power plant with a 1492 MW capacity. Świerkle is in the administrative district of Gmina Dobrzeń Wielki, within Opole County, Opole Voivodship. It is a village with 520 inhabitants. Point three, labelled P3 (50°12ʹ33.46ʹʹ N; 19°28ʹ28.77ʹʹ E), is situated in a rural site of Czyżówka village in the surroundings of a power plant with a capacity of 786 MW. Czyżówka is a village in the administrative district of Gmina Trzebinia, within Chrzanów County, Lesser Poland Voivodship. The village has a population of 702 inhabitants. Point four, labelled P4 (50°13ʹ48.90ʹʹ N; 19°13ʹ24.45ʹʹ E), is situated in the suburb of Jaworzno city near a power plant with an installed capacity of 1345 MW. Jaworzno is a city near Katowice with a population of 95,500 citizens.

In the studied area, a large amount of APM is generally associated with local pollutant sources. At all of the sampling sites, major pollutant sources are power plants including transportation and deposition of coal and fly ash, industrial activities, combustion of coal and wood for domestic cooking purposes and vehicle transport. Additionally, points P1 and P2 are under the influence of soil resuspension from agriculture activities and vehicle traffic on unpaved roads. Meanwhile, at points P3 and P4, there is an impact of urban traffic from neighbouring cities (Katowice, Jaworzno).

### 2.2. Sampling Method

The PM1 samples were collected at sites P1–P4 near four working power plants. The PM1 samples were collected using a three-stage impactor—Dekati^®^ PM10 impactor (Finland) with a flow rate of 30 dm^3^/min. The cut-off diameters of the impactor stages were 10, 2.5 and 1 µm. The samples with a diameter above 1 µm were collected on 25 mm Nuclepore membranes (Whatman International Ltd., Maidstone, UK). The PM1 was collected employing 47 mm Teflon filters (Pall International Ltd., New York, NY, USA). The average volumes of air aspired by the filters was approx. 300 m^3^. Blank filters were stored in the sampling area. According to the manufacturer’s information, the impactor is characterized by uncertainties below 2.8%. The inlet tube was installed 1.5 m above the ground, which is important for human exposure. The membranes and filters were conditioned before and after sampling (temperature 20 ± 1 °C, relative humidity 50% ± 5%) for 48 h and weighted with a microbalance precision of 1 μg (MXA5/1, RADWAG, Poland). The separated submicron particles (PM1) were determined by dividing the mass by the air volume (µg/m^3^).

### 2.3. Fractionation Analysis

The evaluation of the chemical forms of the trace elements in the PM1 samples that were collected in the surroundings of individual four working power plants was determined by applying the sequential extraction scheme presented in Table 1. A chemical fractionation of the trace elements applied the procedure of leaching, based on the scheme of [24] and modified by [22,25].

**Table 1:** Fractionation scheme according to [22,23].

Fraction	Reagent	Experimental Conditions
Fraction 1 (F1)	15 cm^3^ H_2_O Milli-Q	3 h shaking (room temperature)
Fraction 2 (F2)	10 cm^3^ NH_2_OH·HCl (0.25 M)	5 h shaking (room temperature)
Fraction 3 (F3)	7.5 cm^3^ H_2_O_2_ (30%) +7.5 cm^3^ H_2_O_2_ (30%) +15 cm^3^ NH_4_AcO (2.5 M)	First evaporation at 95 °C until near dryness. Second evaporation at 95 °C until near dryness. Shaking 90 min (room temperature).
Fraction 4 (F4)	10 cm^3^ (HNO_3_:HCl:HClO_4_) (6:2:5)	5 h shaking (room temperature)

The solutions that were obtained from the four extraction steps (F1–F4) of the PM1 samples were filtered by the DigiFILTER system, PerkinElmer (0.45 µm). Then, in every fraction, the contents of As, Cd, Co, Cr, Hg, Mn, Ni, Pb, Sb and Se were determined. The total content of each trace element was calculated as the sum of the four extraction steps [21,22].

Regarding the four sessions at each site (P1–P4), the four extracted fractions and the blank filters, the total number of samples was 280. In every sample, 10 trace elements (As, Cd, Co, Cr, Hg, Mn, Ni, Pb, Sb and Se) were quantified.

### 2.4. Chemical Analysis and Quality Control

The concentrations of the trace elements (As, Cd, Co, Cr, Hg, Mn, Ni, Pb, Sb and Se) for each sample were carried out by inductively coupled plasma mass spectrometry (ICP-MS NexION 300D, PerkinElmer, Inc. Waltham, MA, USA).

The instrument is equipped with a multiplier collector and a radio frequency power of 3 kW was applied to the plasma. The standard operational conditions of this instrument are a coolant Ar gas flow rate of 18 dm^3^/min, an auxiliary Ar gas flow rate of 1.2 dm^3^/min, and a nebulizer Ar gas flow rate of 0.96 dm^3^/min. The sample flow rate is 0.5 cm^3^/min. The detection limits for all elements are based on three standard deviations of blanks (*n* = 10) which are listed in Table 2.

**Table 2:** The detection limits of the analysed elements obtained by inductively coupled plasma mass spectrometry (ICP MS).

Isotope	Limit of Detection (LOD) (μg/dm^3^)
^75^As	0.120
^111^Cd	0.034
^59^Co	0.102
^53^Cr	0.245
^200^Hg	0.083
^55^Mn	0.057
^60^Ni	0.325
^206^Pb	0.083
^121^Sb	0.032
^82^Se	0.340

All of the samples were measured in tenfold repeats. Certified multi-element standards 1000 µg/cm^3^ (CertPUR ICP multi-element standard solution VI for ICP-MS) were used as the calibration solution to determine ^75^As, ^111^Cd, ^59^Co, ^53^Cr, ^200^Hg, ^55^Mn, ^60^Ni, ^206^Pb, ^121^Sb and ^82^Se. The concentrations of the trace elements that were contained in PM1 were presented as ng/m^3^ and µg/g. Furthermore, to check the accuracy and precision of the extraction protocol, the European Reference Material ERM^®^-CZ120 and Standard Reference Material SRM 1648a (National Institute of Standards and Technology, USA) were used. The blank filters were also analysed and subtracted from the exposed filters. The sample results were corrected by the average of the blank concentrations. Blank reagents were also taken through each complete procedure. The recovery percentages obtained using the European Reference Material ERM^®^-CZ120 and Standard Reference Material SRM 1648a (National Institute of Standards and Technology, USA) were As (111% for ERM^®^-CZ120 and 96% for SRM 1648a), Cd (97% and 105%), Co (108% and 97%), Cr (103% and 94%), Mn (106% and 100%), Ni (107% and 102%), Pb (107% and 105%), and Sb (99% and 91%). These results indicate a good agreement between the measured and the certified values. The chosen Reference Materials did not include two elements: Hg and Se.

### 2.5. Statistical Analyses

All of the statistical analyses, including a univariate and multivariate analysis, as well as a principal component analysis, were performed using the statistical package, Statistica 10 (StatSoft, Tulsa, OK, USA). Non-parametric tests were undertaken to confirm the parametric results—that is, the corresponding non-parametric tests led to the same conclusions of significance/non-significance as the parametric tests. Throughout the study, a *p*-value of <0.05 was considered to indicate statistical significance.

## 3. Results and Discussion

### 3.1. PM1 Comparison between the Different Sampling Sites

The variability of the PM1 was examined at the four locations in the surroundings of working power plants. In order to compare the concentrations of the trace elements, the results of the total content in PM1 are presented in Table 3. As can be seen (Table 3), the highest average concentration level of PM1 was 12.78 µg/m^3^ in P1. Meanwhile, in the other sampling points, the average PM1 concentrations were at similar levels from 8.13 to 8.74 µg/m^3^. Table 3 contains the sum of the four fractions that represent the total metal concentration in the submicron particles. The range of minimum and maximum concentrations is also presented. The highest concentration among the trace elements collected at all of the sites revealed to be As and Cr (elements carcinogenic to humans) and Pb (probably carcinogenic to humans). In comparison to the average total content of carcinogenic, the probably carcinogenic and toxic elements decrease in the order of As > Cr > Pb > Mn > Se > Ni > Sb > Cd > Hg > Co. Nowadays, worldwide, there is no regulation concerning a submicron particulate PM1 standard for ambient air. Atmospheric particles <1 µm are formed by primary particles resulting from combustion. This particle size fraction consists of a nucleation mode (from particles combustion engine vehicles) and accumulation mode (photochemical smog particles and combustion). Therefore, the existing ambient air quality standards that are restricted to PM2.5 and PM10 fractions, which are generated by mechanical processes, are unable to effectively control submicron particles [26]. The different impact of aerosol sources on PM2.5 and PM1 was also highlighted and show that PM1 can provide a better estimation of anthropogenic particles [27].

**Table 3:** The total mass concentration and concentration range of elements at P1–P4 sites.

Elements	Average Concentration, ng/m^3^								Concentration Range, ng/m^3^			
	P1	SD	P2	SD	P3	SD	P4	SD	P1	P2	P3	P4
As	28.27	0.40	28.38	0.17	18.54	0.44	18.96	0.23	27.72–28.65	28.15–28.57	17.91–18.91	18.64–19.12
Cd	0.46	0.01	0.52	0.06	0.39	0.16	0.76	0.16	0.45–0.47	0.44–0.57	0.17–0.55	0.54–0.90
Co	0.16	0.003	0.16	0.003	0.03	0.00	0.03	0.01	0.16–0.17	0.16–0.17	0.02–0.03	0.03–0.04
Cr	21.47	0.23	21.49	0.09	8.47	0.19	8.60	0.04	21.29–21.80	21.37–21.58	8.20–8.61	8.56–8.64
Hg	0.52	0.01	0.52	0.00	0.07	0.07	0.02	0.00	0.51–0.53	0.52–0.52	0.02–0.18	0.02–0.02
Mn	4.74	0.96	5.08	0.73	3.27	0.82	5.71	1.52	3.73–6.03	4.43–6.10	2.14–4.07	4.21–7.80
Ni	2.34	0.08	2.07	0.10	2.61	3.10	0.37	0.12	2.26–2.45	1.98–2.21	0.34–6.99	0.25–0.54
Pb	7.25	0.36	9.91	0.73	11.21	2.69	18.09	5.93	6.78–7.64	9.19–10.92	7.41–13.20	13.10–26.42
Sb	0.99	0.63	0.56	0.09	0.44	0.17	1.67	0.43	0.54–1.89	0.47–0.67	0.20–0.57	1.14–2.18
Se	7.11	0.07	7.71	0.35	1.23	0.53	1.37	0.27	7.01–7.19	7.22–8.02	0.78–1.97	1.12–1.74
PM1, µg/m^3^	12.78	3.16	8.68	4.18	8.74	1.62	8.13	4.77	8.80–17.56	4.24–13.96	6.03–10.29	4.31–11.62

**Figure 2:**
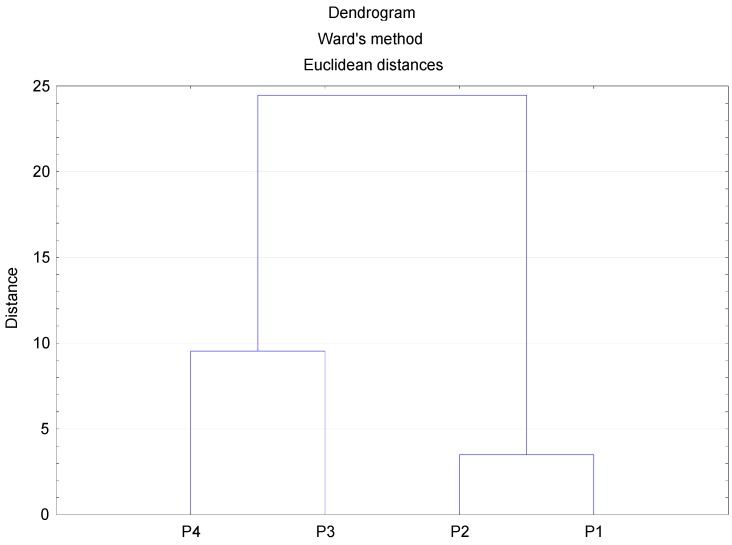
Dendrogram obtained in a cluster analysis for the average PM1 concentration from four sites in the surroundings of working power plants.

According to a cluster analysis that was obtained in STATISTICA environment, using Ward’s method (Figure 2), the sampling points located in the surroundings of the working power plants were grouped by the average PM1 concentrations. The first group obtains sites P3 and P4 and the second P1 and P2, respectively.

In general terms, the grouping obtained with the cluster analysis points to similar emission sources. As mentioned in Section 2.1. (Sampling sites), the first group (points P1 and P2) is mainly under the influence of agriculture activities. Meanwhile, the second group (points P3 and P4) is mainly under the influence of urban sources.

### 3.2. Distribution of the Elements by the Sequential Extraction in PM1

Information about the mass and total content of the trace elements in PM1 is necessary but insufficient in order to evaluate the overall pollution and hazard levels. This is because the effect of the trace elements in the environment and humans depends on the chemical form in which the elements are bound.

**Figure 3:**
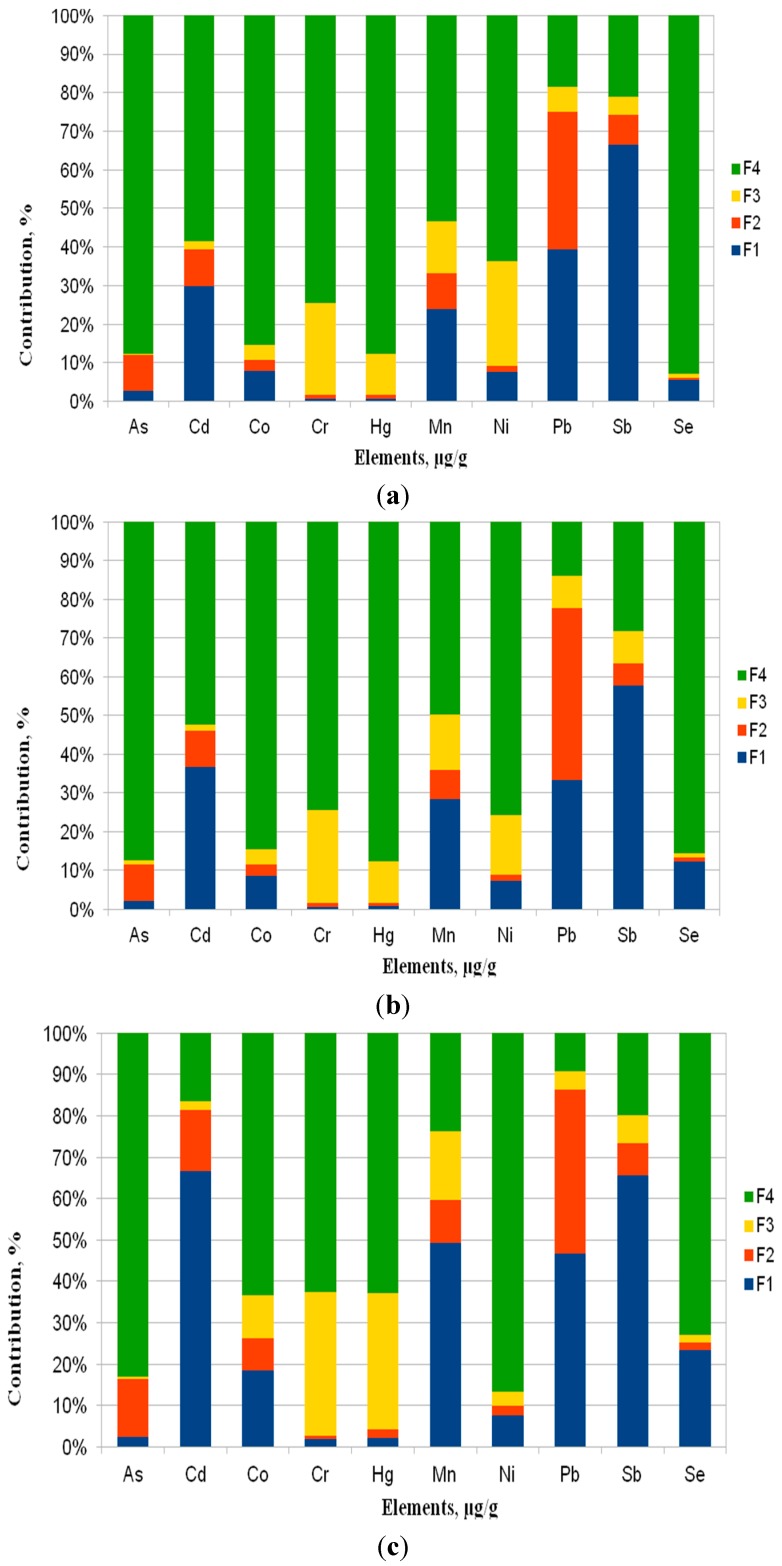

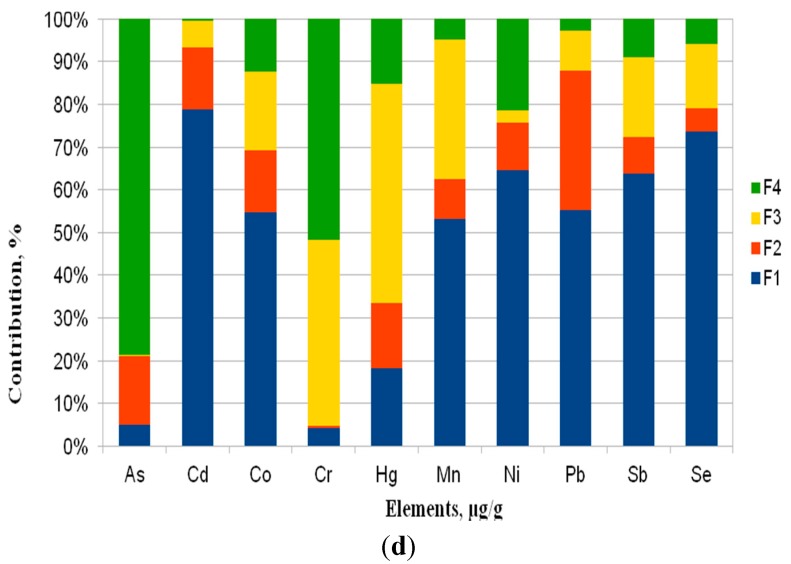
Chemical form distribution of trace elements as a percentage of the elemental concentration in PM1 collected at site (**a**) P1; (**b**) P2; (**c**) P3; (**d**) P4.

Fractionation data (Figure 3) for the elements in PM1 are shown as the percentage of the sum of concentrations found in the four steps by the sequential extraction procedure. These figures indicate how much of each trace element is present in each fraction. The distribution of trace elements (Figures 3) points to the similarity of locations P1 and P2, which confirms the hypothesis of the dendrogram (Figure 2). On the other hand locations P3 and P4 are characterized by a clearly different distribution for most of the elements and, particularly, for Se, Ni and Hg. This probably point to different sources of these trace elements in the selected area.

Generally, the environmentally highly mobile character of the elements (Fraction 1) in the PM1 samples that were collected in the surroundings of the working power plants decreases in the following order: Sb > Cd > Pb > Mn > Se > Co > Ni > As > Cr = Hg. With regard to the mobile elements (Fraction 2), a decrease in the order: Pb > Cd = As > Mn > Sb > Co > Ni > Se = Hg = Cr was observed. For the less mobile Fraction 3, the elements decrease in the order of: Cr > Mn > Hg = Ni > Sb > Pb > Co > Cd > Se > As. While the elements in the not mobile Fraction 4 decrease in the order of: As > Hg > Se > Co > Ni > Cr > Mn > Cd > Sb > Pb.

In our study, the highly mobile elements—and thus particularly harmful to humans (fraction F1)—were Cd, Mn, Sb and Pb, which also revealed the highest distribution in mobile fraction (F2). While As, Co, Cr, Hg, Ni and Se were mainly present in the not mobile fraction (F4).

In the water-soluble fraction (F1), the average substantial percentage of Mn, Pb, Cd and Sb was 39%–64%. Furthermore, 37% of Pb and 1%–12% of the other elements were in mobile fraction (F2). The less mobile (F3) were 1%–28% of all of the elements and 69%–85% of Cr, Ni, Co, Se, Hg and As were the most abundant in the not mobile fraction (F4). Currently, there are no data available to compare the chemical fractionation of trace elements in submicron particles (PM1) that were collected in the surroundings of power plants fired with hard coal. Similar studies were presented by Manousakas *et al.* [5,28]. However they only investigated the elemental composition of water-soluble and acid-soluble fractions of PM2.5 samples collected in a Greek city located closely to lignite power plants. According to different emission profiles of lignite-fired power plants and related higher adverse environmental effects of Greek power plants, the results cannot be compared with our study (hard coal-fired power plants).

In the study of Canepari *et al.* [29], which was conducted during a five-year field research in the peri-urban area of Ferrara, in northern Italy, a chemical fractionation procedure has shown that some of the toxic elements (As, Cd, Tl, V) are mostly present in fine fraction (PM2.5) and as soluble chemical species. Thus, they are particularly accessible to environmental and biological systems. In our study, the most abundant elements in water-soluble fractions of submicron particulate matter (PM1), which are particularly dangerous to the environment, were Cd (carcinogenic to humans), Pb (probably carcinogenic to humans) and Mn and Sb (toxic elements). Meanwhile, As (carcinogenic to humans) was mostly (85%) determined in the not mobile fraction (F4). A similar property of As was revealed in atmospheric aerosols (APM), collected in a Hungarian moderately polluted city and a regional background sampling site. Here, the percentages of As in the not mobile fraction (F4) was from 88% to 94% [30]. Research on PM1 collected in a Hungarian meteorological background station [31] also revealed that as mainly occurs in environmentally immobile fractions (77%).

In this study, fractionation data of the sequential extraction procedure show that Pb compounds in every location are present in the environmentally highly mobile and mobile fractions. The average sum of F1 and F2 was 83%. Precisely in P1, it was 39 and 36%, 33% and 44% in P2, 47 and 40% in P3 and 55% and 33% in P4, respectively. In another study [21], the sum of the first two mobile fractions for Pb fell in the interval 70%–94%, constituting a high source of environmental pollution in PM10. This behaviour indicates that likely diverse sources of Pb compounds are characterized by different solubility.

### 3.3. Enrichment Factor (EF) Analysis

**Figure 4:**
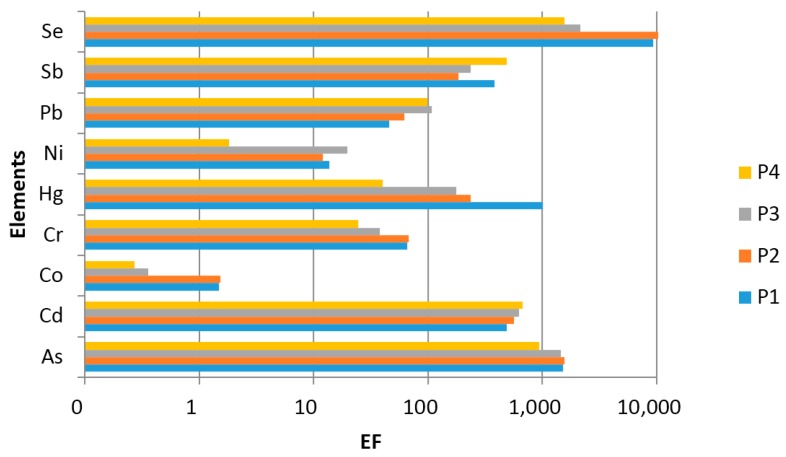
Enrichment factors of elements with Mn as the reference element.

Major and trace elements in particulate matter can be classified as natural (Na, Mg, K, Ca, Si, Al, Mn, *etc.*) or anthropogenic (V, Cr, Mn, Ni, Cu, Zn, Cd, Pb, *etc.*) [32]. In order to have a preliminary indication on the contribution of anthropogenic emissions, for each element, we calculated the average enrichment factor according to the following equation:

(1) $$EF_{x}=\frac{{\left(c_{x}/c_{ref}\right)}_{PM_{1}}}{{\left(c_{x}/c_{ref}\right)}_{crust}}$$

where *c_x_* and *c_ref_* are the total content of the element *x* and the reference element, and (*c_x_*/*c_ref_*)*_PM1_* and (*c_x_*/*c_ref_*)*_crust_* are the proportions of these concentrations in the PM1 and in the upper continental crust [33,34], respectively. Si, Al, or Fe are generally used as the reference elements for the main source of the Earth’s crust composition. Other elements can be used such as Mn [35,36], Sr [37] or Ti [38]. In this study, Mn has been used as the reference element for the Earth’s crust. Consequently, EF_Mn_ = 1. As an indication on the crustal and non-crustal sources of PM1 collected in the surroundings of power plants, the enrichment factor of each element was calculated (Figure 4). EFs can provide information that could be used for the discussion of the PCA results. However, the chemical composition of the geological environment of Upper Silesia in Poland does not necessarily have the same chemical composition as the average Upper Continental Crust as estimated by Wedepohl [34]. The origin of Mn is not only geogenic, thus the EFs values may be underestimated.

According to the results of the EF analysis, the trace elements in PM1 were divided into two groups. Group 1a with EFs ~1 includes Co (at all sites). This element is considered to originate from the crust.

Group 1b includes Ni with EFs between 2 and 20. Ni can originate from soil or road dust resuspension and from mixed anthropogenic and geogenic sources.

Group 2a with 10 < EFs < 100 (moderately enriched) includes Cr and Pb (at all sites), while Hg is only included at site P4.

Group 2b with EFs > 100 (highly enriched) includes As, Cd, Sb and Se (at all sites), while Hg at sites P1, P2 and P3.

The elements assigned to groups 1a, 1b as well as 2a, 2b are believed to have mixed (anthropogenic and geogenic) and anthropogenic origin, respectively. High enrichment factors show that, in our sampling sites, there are relevant emission sources that determine the metal loading in the ambient air. The average EFs of these elements in the surroundings of four individual working power plants indicate that traffic and coal combustion, as well as other industrial activities based on coal, might be responsible for a higher enrichment degree of anthropogenic elements. However, it should be mentioned that EFs are strongly dependent on particle size. In general, high EF values reveal elements that are mostly distributed in fine particles (PM2.5). This is a result of their long residence time in the atmosphere, as well as their enrichment with gas-phase components [39].

### 3.4. Principal Component Analysis (PCA)

PCA with Varimax rotation was applied for the identification of the possible pollutant sources of trace elements in the PM1 samples that were determined in each fraction, namely Fraction 1, Fraction 2, Fraction 3 and Fraction 4. Although there are no well-defined rules for the number of factors to be retained, usually either factors that are meaningful or factors with eigenvalues larger than unity are retained. In this study, three principal components (PCs) were extracted. Component loadings lower than 0.20 are suppressed. Loadings larger than 0.5 (in absolute values) are considered to be important.

For all of the fractions (F1–F4), the Pearson’s correlation matrix between the trace elements in PM1 samples collected in the surroundings of four power plants (P1–P4) was calculated. Table 4 presents the exemplary correlation matrix for fraction 3 (F3). The possible sources around the sampling sites can be qualitatively identified from the single correlation coefficients. Based on a strong correlation (*r* ≥ 0.75) two groups of variables can be distinguished: the first consisting of Cd, Mn, Pb, Sb and Se and the latter consisting of Co, Cr and Ni. Hence, it would hypothesize a different source for groups 1 and 2.

**Table 4:** Correlation coefficients between the different trace elements for Fraction 3 collected in the surroundings of four power plants.

Element	As	Cd	Co	Cr	Hg	Mn	Ni	Pb	Sb	Se
As	1.00									
Cd	−0.23	1.00								
Co	0.46	−0.40	1.00							
Cr	0.51	−0.43	**0.96**	1.00						
Hg	0.11	−0.36	0.42	0.26	1.00					
Mn	−0.16	**0.88**	−0.51	−0.45	−0.53	1.00				
Ni	0.23	−0.39	**0.80**	**0.87**	0.19	−0.36	1.00			
Pb	−0.15	**0.80**	**0.92**	−0.26	−0.42	**0.84**	−0.30	1.00		
Sb	−0.21	**0.87**	−0.36	−0.40	−0.42	**0.87**	−0.35	**0.96**	1.00	
Se	−0.12	**0.75**	−0.20	−0.22	−0.23	**0.77**	−0.20	**0.93**	**0.92**	1.00

*r* ≥ 0.75 is bold.

The PCA analysis has been performed in three steps. The first step included the execution of four PCA analyses—separately for each fraction (Fractions 1–4). This step was applied to determine the associations between the elements generated by diverse emission sources (extracted in each fraction). The results of single PCA analysis are presented in supplementary material (Tables S1–S4).

The second step included selection of the highest factor loadings as relevant factors (PC1, PC2 or PC3). For example, in Fraction 1 selenium (Se) had the highest factor loading for third principal component (PC3); for Fraction 2 the highest factor loading was for PC2, while for Fraction 3 and Fraction 4, it was PC1 (Table 5).

**Table 5:** The total explained variance.

Element	Fraction 1	Fraction 2	Fraction 3	Fraction 4 *
Number of PCs	3	3	3	2
As	PC2	PC1	PC2	PC1
**Cd**	**PC1**	**PC2**	**PC1**	**PC1**
*Co*	*PC2*	*PC3*	*PC2*	*PC1*
Cr	PC1	PC1	PC2	PC1
Hg	PC1	PC1	PC3	PC1
**Mn**	**PC1**	**PC2**	**PC1**	**PC1**
*Ni*	*PC2*	*PC3*	*PC2*	*PC2*
**Pb**	**PC1**	**PC2**	**PC1**	**PC1**
**Sb**	**PC1**	**PC2**	**PC1**	**PC2**
**Se**	**PC3**	**PC2**	**PC1**	**PC1**
Sum of variance, %	82.1	84.0	86.1	92.8

* correlation matrix is singular; Bold, italic and underlined entries indicate first, second and third factor, respectively.

The third step included grouping of elements, according to the highest similarity in classification performed in second step. For example Cd and Sb have been classified into one group because these elements have the similar series of PCs (PC1–PC2–PC1–PC1 and PC1–PC2–PC1-PC2, respectively).

Mobile fractions F1–F3 were determined by three factors (PC1, PC2 and PC3) with a total variance of 82.1%, 84.0% and 86.1%, respectively. On the other hand, the immobile fraction (F4) was determined by two factors (PC1 and PC2) with a total variance of 92.8%. The dominant factor (with the highest PCA loading) of each element has been presented in Table 5. According to particular distribution of each element, three possible components were identified to assess the sources responsible for the observed pollution levels.

The PCA analysis showed the accuracy of the clustering of elements for all of the fractions (Fraction 1, Fraction 2, Fraction 3 and Fraction 4). Bold, italic and underlined entries indicate the first, second and third component, respectively.

The first component (bold entries), which is mainly classified for factor PC1, is loaded with Cd, Mn, Pb, Sb and Se, which represent vehicular emissions. Elements like Sb, Se, Cd and Pb occur in car exhaust and non-exhaust (brake wear) and Mn (tyre wear) [40,41,42,43]. Mn and Sb may represent the re-suspension of surface road dust that comes from vehicle exhaust emissions and abrasion of automobile tire [35,44,45]. Although Pb is no longer added to petrol, it is found as a trace element in various fuels. Moreover, it still persists in road dust from earlier vehicular exhaust emission due to its longer residence time in the environment [46]. The Manganese tricarbonyl compound is used as an additive in unleaded petrol to enhance automobile performance. These compounds contain organ manganese, which has toxicological significance [44,47]. Sb, Cd, and Pb occur in car exhausts [43].

Cd, Pb, Sb and Se also agree well with the group of elements with a high enrichment factor, which indicates different grades of enrichment due to anthropogenic sources. This relation had also been noted in the work [48]. While other research [49] has reported that the elements with high water solubility were also characterized by a large EF values. In our study, water-soluble elements, namely Cd, Mn, Pb and Sb, originate from the same group, according to PCA analysis. It should be underlined that the trace elements in soluble fractions are adsorbed on particle surfaces and are almost completely bioavailable [50]. Thus, our first source attributed to vehicular emission plays a decisive role for the assessment of atmospheric pollution and its hazards to human health at the examined sites.

The second component (italic entries), which is mainly classified for factor PC2, is characterized by Ni and Co. Presence of these metals in one factor according to EF values <20 provides an indication of possibly mixed anthropogenic and crustal sources, namely emissions of biomass burning and waste incineration, as well as from soil or road dust resuspension. Oil combustion is the important source of Ni [13,50,51,52]. Meanwhile, soil dust is a characteristic source of Co [53].

The third component (underlined entries) that is mainly classified for factor PC3 includes As, Cr and Hg. This suggests the presence of anthropogenic sources, like coal combustion processes, in large power plants and fuel combustion to produce heat and electricity [13,52]. It can also be well associated with the domestic fossil fuel burning (oil and coal) [32], which is used for every day cooking. In these residential regions, this is the main source of these trace elements.

**Figure 5:**
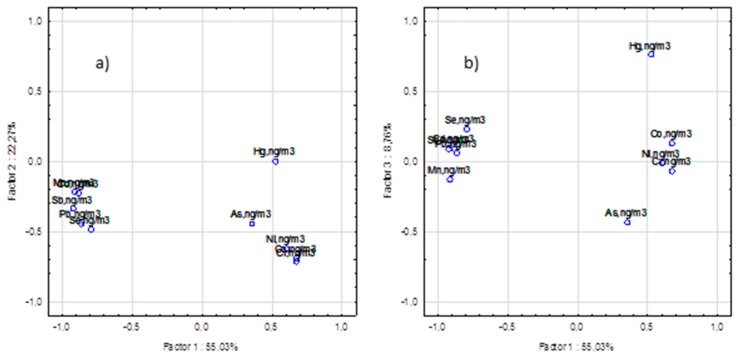
The scatterplot of the loading factors for Fraction 3. (**a**) for the first and second principal components; (**b**) for the first and third principal components projection of variables according to the principle component analysis (PCA).

Figure 5 is an exemplary visualization of the apportionment of the proposed emission sources according to the principle component analysis (PCA) of Fraction 3. Figure 5a shows the scatterplot of the first and second principal components, while Figure 5b shows the scatterplot of the first and third principal components.

## 4. Conclusions

Globally, coal combustion is one of the main power supplies. The fate of trace elements in coal-fired power plants that are hazardous to humans requires scientific interest. This study investigated the characteristics of submicron particles, which were collected at the surroundings of hard coal-fired power plants in southern Poland. These included the concentration, trace elements composition and mobility. This study also investigated source identification. The average concentration levels of PM1 were from 8.13 to 12.78 µg/m^3^.

In order to evaluate the overall pollution and hazard levels the information on the total content of trace elements in PM1 is necessary but insufficient. Based on sequential chemical extraction, the four fractions were separated (highly mobile—F1; mobile—F2; less mobile—F3 and not mobile—F4). The highly mobile elements (F1), thus particularly harmful to human, were Cd, Mn and Sb. Only Pb revealed high distribution in highly mobile and mobile fractions (F1 and F2). While As, Co, Cr, Hg, Ni and Se were present mainly in not mobile fraction (F4).

The enrichment factors for As, Cd, Sb and Se were above 100, which indicated their leading role in anthropogenic sources. Moderately enriched were Cr, Hg and Pb. According to EF < 20, Ni can originate from soil or road dust resuspension, as well as mixed anthropogenic and geogenic sources. Meanwhile, Co is considered to mainly originate from crust.

Source identification through PCA was performed first separately for each fraction then for all fractions*.* Based on the highest PCA loading, three major sources of trace elements were identified: Vehicular emissions, mixed anthropogenic and crustal sources and fossil fuel combustion.

The results also underscore the importance of future investigations into submicron and fine particle-bound elements in PM characteristic for industrial sources (e.g., power and coking plants) for improved exposure-related health-risk assessments.

## Supplementary Files

Supplementary File 1
