# Supplementary material for: Trace Elements Speciation of Submicron Particulate Matter (PM1) Collected in the Surroundings of Power Plants
Source: Int J Environ Res Public Health. 2015 Oct 16;12(10):13085–103. doi: 10.3390/ijerph121013085 (PMC4627018; doi:10.3390/ijerph121013085)
Supplement: Supplementary File 1 [file ijerph-12-13085-s001.pdf]

Trace Elements Speciation of Submicron Particulate Matter (PM1) Collected in the Surroundings of Power Plants

Table S1. Principal component loadings and variance explanation for Fraction 1.

Element	PC1	PC2	PC3
As	0.331	0.680	0.109
Cd	0.760	0.291	0.467
Co	0.234	0.655	0.610
Cr	0.834	0.308	0.226
Hg	−0.670	0.445	−0.305
Mn	0.768	0.376	0.470
Ni	0.259	0.883	0.040
Pb	0.681	0.548	0.381
Sb	0.793	0.236	−0.057
Se	0.231	0.021	0.928
% variance	36.8	25.5	19.8

Component loadings lower than 0.20 are suppressed. Loads larger than 0.5 (in absolute values) are in bold.

Table S2. Principal component loadings and variance explanation for Fraction 2.

Element	PC1	PC2	PC3
As	0.965	0.056	0.226
Cd	0.641	0.690	−0.169
Co	−0.044	0.380	0.794
Cr	−0.989	−0.123	−0.064
Hg	−0.985	−0.143	−0.007
Mn	0.025	0.812	0.214
Ni	0.225	−0.128	0.882
Pb	0.467	0.775	0.025
Sb	0.207	0.834	0.169
Se	−0.227	0.658	−0.345
% variance	36.5	30.6	16.9

Component loadings lower than 0.20 are suppressed. Loads larger than 0.5 (in absolute values) are in bold.

Table S3. Principal component loadings and variance explanation for Fraction 3.

Element	PC1	PC2	PC3
As	−0.144	0.596	0.375
Cd	0.864	−0.279	0.102
Co	−0.159	0.934	−0.234
Cr	−0.188	0.968	−0.039
Hg	−0.281	0.220	−0.849
Mn	0.844	−0.289	0.318
Ni	−0.165	0.848	−0.086
Pb	0.956	−0.089	0.138
Sb	0.955	−0.208	0.117
Se	0.958	−0.028	−0.036
% variance	55.0	22.3	8.8

Component loadings lower than 0.20 are suppressed. Loads larger than 0.5 (in absolute values) are in bold.

Table S4. Principal component loadings and variance explanation for Fraction 4.

Element	PC1	PC2
As	0.994	0.105
Cd	0.990	0.135
Co	0.990	0.135
Cr	0.991	0.128
Hg	0.990	0.135
Mn	0.990	0.133
Ni	−0.048	0.935
Pb	0.721	0.667
Sb	0.365	0.568
Se	0.989	0.101
% variance	75.3	17.5

Component loadings lower than 0.20 are suppressed. Loads larger than 0.5 (in absolute values) are in bold.

© 2015 by the authors; licensee MDPI, Basel, Switzerland. This article is an open access article distributed under the terms and conditions of the Creative Commons Attribution license (http://creativecommons.org/licenses/by/4.0/).
